# Cognitive functions and metabolic syndrome in childhood-onset systemic lupus erythematosus

**DOI:** 10.1186/1546-0096-12-S1-P323

**Published:** 2014-09-17

**Authors:** Nailu Sinicato, Mariana Postal, Bruna Bellini, Paula Fernandes, Roberto Marini, Simone Appenzeller

**Affiliations:** 1 Medicine, State University Of Campinas, Campinas, Brazil; 2 Sports, State University Of Campinas, Campinas, Brazil; 3 Pediatrics, State University Of Campinas, Campinas, Brazil

## Introduction

Childhood-onset systemic lupus erythematosus (cSLE) have a high prevalence cognitive involvement and inflammatory mechanisms and autoantibodies were hypothesized to be involved in its pathogenesis. The underlying factor for an association between the metabolic syndrome (MetS) and cognitive decline might be a subclinical inflammation.

## Objectives

To determine if cognitive functions are impaired in cSLE patients with MetS.

## Methods

We performed a cross sectional study of 63 consecutive cSLE patients and 63 healthy age and sex matched controls. All individuals were assessed for anthropometric and MetS features according to International Diabetes Federation (IDF) criteria. Neurological manifestations were analyzed according to the ACR classification criteria. Cognitive evaluation was performed in all participants using Wechsler Intelligence Scale for children (WISC-III) and Wechsler Intelligence Scale for adults (WAIS), according to age and validated in Portuguese. Mood disorders were determined through Becks Depression and Anxiety Inventory in all participants. SLE patients were further assessed for clinical and laboratory SLE manifestations, disease activity [SLE Disease Activity Index (SLEDAI)], damage [Systemic Lupus International Collaborating Clinics/American College of Rheumatology Damage Index (SDI)] and current drug exposures. Total dose of corticosteroids and other immunosuppressant medications used since the onset of disease were calculated by data obtained by careful review of the medical charts.

## Results

We observed higher hip circumference (p=0.030), waist-to-hip ratio (p<0.001) and hypertriglyceridemia (p=0.005) in cSLE patients. Controls had a higher height (p=0.003) and higher levels of HDL-c (p=0.004). MetS was present in 11 (17.4%) cSLE and in no control. Cognitive dysfunction was observed in 32 (50.8%) cSLE patients. We observed an inverse correlation with height and corticosteroid total dose adjusted by weight in cSLE patients (-0.285; p=0.022). Rey complex picture on memory subtest was correlated with body mass index (r=-0.249; p=0.05) and hypertriglyceridemia (r=0.282;p=0.028). Total cholesterol levels was correlated with Boston naming test (r=-0.258;p=0.047).

## Conclusion

MetS was observed in 18% of our cohort and not associated with worse cognitive performance. However, features of MetS, such as total cholesterol, hypertriglyceridemia and obesity can influence some cognitive functions in cSLE.

## Disclosure of interest

N Sinicato: None declared, M Postal: None declared, B Bellini: None declared, P Fernandes: None declared, R Marini: None declared, S Appenzeller Grant / Research Support from: FAPESP (2008/02917-0, 2011/03788-2, 2013/09480-5) CNPQ (300447/2009-4 and 471343/2011-0; 302205/2012-8; 473328/2013-5)

